# EMNGly: predicting N-linked glycosylation sites using the language models for feature extraction

**DOI:** 10.1093/bioinformatics/btad650

**Published:** 2023-11-01

**Authors:** Xiaoyang Hou, Yu Wang, Dongbo Bu, Yaojun Wang, Shiwei Sun

**Affiliations:** Key Laboratory of Intelligent Information Processing, Institute of Computing Technology, Beijing 100190, China; University of Chinese Academy of Sciences, Beijing 100049, China; Syneron Technology, Guangzhou 510000, China; Key Laboratory of Intelligent Information Processing, Institute of Computing Technology, Beijing 100190, China; University of Chinese Academy of Sciences, Beijing 100049, China; College of Information and Electrical Engineering, China Agricultural University, Beijing 100083, China; Key Laboratory of Intelligent Information Processing, Institute of Computing Technology, Beijing 100190, China; University of Chinese Academy of Sciences, Beijing 100049, China

## Abstract

**Motivation:**

N-linked glycosylation is a frequently occurring post-translational protein modification that serves critical functions in protein folding, stability, trafficking, and recognition. Its involvement spans across multiple biological processes and alterations to this process can result in various diseases. Therefore, identifying N-linked glycosylation sites is imperative for comprehending the mechanisms and systems underlying glycosylation. Due to the inherent experimental complexities, machine learning and deep learning have become indispensable tools for predicting these sites.

**Results:**

In this context, a new approach called EMNGly has been proposed. The EMNGly approach utilizes pretrained protein language model (Evolutionary Scale Modeling) and pretrained protein structure model (Inverse Folding Model) for features extraction and support vector machine for classification. Ten-fold cross-validation and independent tests show that this approach has outperformed existing techniques. And it achieves Matthews Correlation Coefficient, sensitivity, specificity, and accuracy of 0.8282, 0.9343, 0.8934, and 0.9143, respectively on a benchmark independent test set.

## 1 Introduction

Glycosylation is a post-translational modification process (Mazola *et al.* 2011) that occurs in cells, wherein sugar molecules, also known as glycans, are chemically added to specific acceptor amino acids—such as asparagine, serine, and threonine—of proteins or lipids (Ohtsubo and Marth 2006). It is estimated that over fifty percent of human proteins undergo glycosylation, a process that occurs ubiquitously across various species and cell types (Zaia 2008, Hart and Copeland 2010, Zhang and Wang 2016).

Glycosylation is the most intricate post-translational modifications. It takes place during protein biosynthesis and has significant effects on the expression, localization, folding, and half-life of secreted and cell surface proteins. Additionally, it participates in critical biological processes like cell adhesion, trafficking, receptor binding, and activation. Furthermore, glycosylation plays an essential role in regulating immune system function and is involved in numerous disease processes (Ohtsubo and Marth 2006, Lederkremer 2009, Varki and Sharon 2009, Aebi *et al.* 2010).

There are four main types of glycosylation: N-linked, O-linked, C-linked, and S-linked. N-linked glycosylation refers to the attachment of a carbohydrate molecule to an amino acid residue on a protein via an N-glycosidic bond. This type of glycosylation occurs in the endoplasmic reticulum and Golgi apparatus. O-linked glycosylation involves the attachment of a carbohydrate molecule to a hydroxyl group on a serine or threonine residue on a protein via an O-glycosidic bond. This type of glycosylation occurs primarily in the Golgi apparatus. C-linked glycosylation involves the attachment of a carbohydrate molecule to a tryptophan residue on a protein via a C-glycosidic bond. This type of glycosylation is rare and has only been observed in certain bacteria and archaea. S-linked glycosylation involves the attachment of a carbohydrate molecule to a cysteine residue on a protein via an S-glycosidic bond. This type of glycosylation is also rare and has only been observed in a few species of bacteria.

N-linked glycosylation is the most common type of glycosylation in eukaryotic cells, accounting for ∼90% of all glycosylation events. It involves the attachment of N-glycans (oligosaccharide) to the amine group (NH2) of asparagine (Asn (N)) within N-X-S/T (S: serine, T: threonine) sequons, where X is any amino acid excluding Proline (Pro). However, not all of these sequons are glycosylated. Some sequons may not be recognized by the glycosylation machinery because the glycosylation is a complex process that can be influenced by various cellular factors and conditions (Gavel and von Heijne 1990, Ben-Dor *et al.* 2004, Kowarik *et al.* 2006, Weerapana and Imperiali 2006).

Experimental techniques, including mass spectrometry (Agarwal *et al.* 1969, Huang *et al.* 2020a,b, Wang *et al.* 2020) and lectin binding (Liu and Wang 2015), can be used to identify the specific positions in the protein sequence that have undergone glycosylation. However, these methods (Sun *et al.* 2018, Ju *et al.* 2019, Wang *et al.* 2019, Wang *et al.* 2021, Huang *et al.* 2022) are expensive and time-consuming, requiring specialized equipment and skilled personnel. To overcome these challenges, computational approaches that utilize machine learning and deep learning algorithms have become increasingly popular and necessary. These techniques can provide results within a few hours, making them a cost-effective and efficient alternative to traditional experimental methods.

Numerous outstanding computational models have been created to predict glycosylation sites. These models can be classified into two categories based on the input information they receive: sequence-based predictors and structure-based predictors.

Sequence-based predictors use various features extracted from the protein sequence, such as amino acid composition, motifs, physicochemical properties, and evolutionary information. Structure-based predictors, on the other hand, rely on the 3D structure of the protein to predict glycosylation sites. These methods use various structural features, including solvent accessibility, secondary structure, and electrostatic potential, to identify potential glycosylation sites.

Several publicly available approaches that use sequence-based features include NetNGlyc (Gupta and Brunak 2001), GlycoPP (Chauhan *et al.* 2012), GlycoEP (Chauhan *et al.* 2013), GlycoMine (Li *et al.* 2015), and others. NetNGlyc tool uses artificial neural networks for N-linked glycosylation prediction. GlycoPP utilizes support vector machine (SVM) for prediction based on binary profile of patterns, composition profile, and PSSM profile of patterns (PPP) for human protein sequences. GlycoEP performs Binary, AAC, PSSM, SS, and ASA coding for large Eukaryotic dataset and uses SVM machine learning technique to predict glycosylation sites. GlycoMine tool uses sequence-based features and utilizes a random forest (RF) classifier to predict N-linked and O-linked glycosylation sites.

To more comprehensively incorporate features related to glycosylation sites, many methods further consider the protein’s three-dimensional structure information (Marino *et al.* 2010, Moremen *et al.* 2012).

GPP (Hamby and Hirst 2008), developed by Hamby and Hirst, uses a combination of secondary structure and sequence-based features to train a random forest classifier. GlycoMine^struct^ (Li *et al.* 2016) uses the random forest for classification and linear SVM for feature selection. SPRINT-Gly (Taherzadeh *et al.* 2019) utilizes deep neural networks and SVM machine learning methods to identify N-linked and O-linked glycosylation sites on large datasets extracted from six human and mouse databases. N-GlyDE uses SVM machine learning. PUStackNGly (Alkuhlani *et al.* 2022) performs feature encoding using Binary, PSSM, and seven other methods, and uses stacking ensemble machine learning to predict glycosylation sites. DeepNGlyPred (Pakhrin *et al.* 2021) is an approach based on deep learning that utilizes a combination of sequence-based features, predicted structural features, and evolutionary information to encode positive and negative sequences in the human proteome dataset. Besides, an approach called LMNglyPred (Pakhrin *et al.* 2023) was developed, which utilizes embeddings from a pre-existing protein language model (pLM).

While the presence of the consensus N-X-[S/T] motif does not always lead to glycosylation (Gavel and von Heijne 1990), it should be noted that most methods for predicting glycosylation sites do not limit themselves to this sequon, except LMNglyPred, NetNGlyc, N-GlyDE, and DeepNGlyPred. This may explain why the predictive performance of approaches tends to overestimate the task of predicting N-linked glycosylation sites, as they simply predict each N in the N-X-[S/T] sequon as a potential glycosylation site. Nonetheless, LMNglyPred, NetNGlyc, N-GlyDE, and DeepNGlyPred are significant contributions to the field as they acknowledge that the N-X-[S/T] sequon is necessary but insufficient for N-linked glycosylation.

While the current models have achieved significant success in predicting glycosylation sites, there are still some issues that need to be addressed to improve the accuracy and reliability of these methods (Alkuhlani *et al.* 2021).

One major issue is the lack of data on glycosylation sites. Glycosylation is a complex process that involves multiple enzymes and pathways, data-lacking can lead to overfitting or underfitting of models, which can affect their predictive performance.

Another area of concern is that most current glycosylation site prediction methods heavily rely on manually extracted features, which can be subjective and incomplete, and may lead to redundancy and a lack of comprehensiveness.

Finally, glycosylation is dependent on context, implying that there is a distant correlation between the amino acid sequence of a protein and the precise locations where glycans are fastened. Nonetheless, the majority of current techniques solely focus on the surrounding sequence locally, neglecting to consider the overall context of the protein sequence.

In this article, we present a new predictor for N-linked glycosylation confined to N-X-[S-T] sequon. Our approach leverages the largest dataset to date for model training, enabling the model to potentially recognize more complex patterns and make more accurate predictions. Specifically, we incorporate a pre-trained language model that has been fine-tuned on a large corpus of protein sequences to predict the probability of glycosylation at each residue position. This approach allows us to leverage the power of transfer learning and increase the accuracy of our predictions on new, unseen data. Structural information are also incorporated in our model to provide information about the environment surrounding the glycosylation site, including the physical and chemical properties of residues (such as charge, hydrophobicity, spatial constraints). By combining these different sources of information, our model is able to make more accurate and robust predictions of glycosylation sites in proteins. Our results indicate that the proposed strategy achieves state-of-the-art performance on N-GlyDE dataset, with values for Matthews Correlation Coefficient (MCC), Sensitivity (SN), Specificity (SP), and ACC of 0.736, 0.70, 0.975, and 0.884, respectively.

## 2 Materials and methods

**Figure 1.**
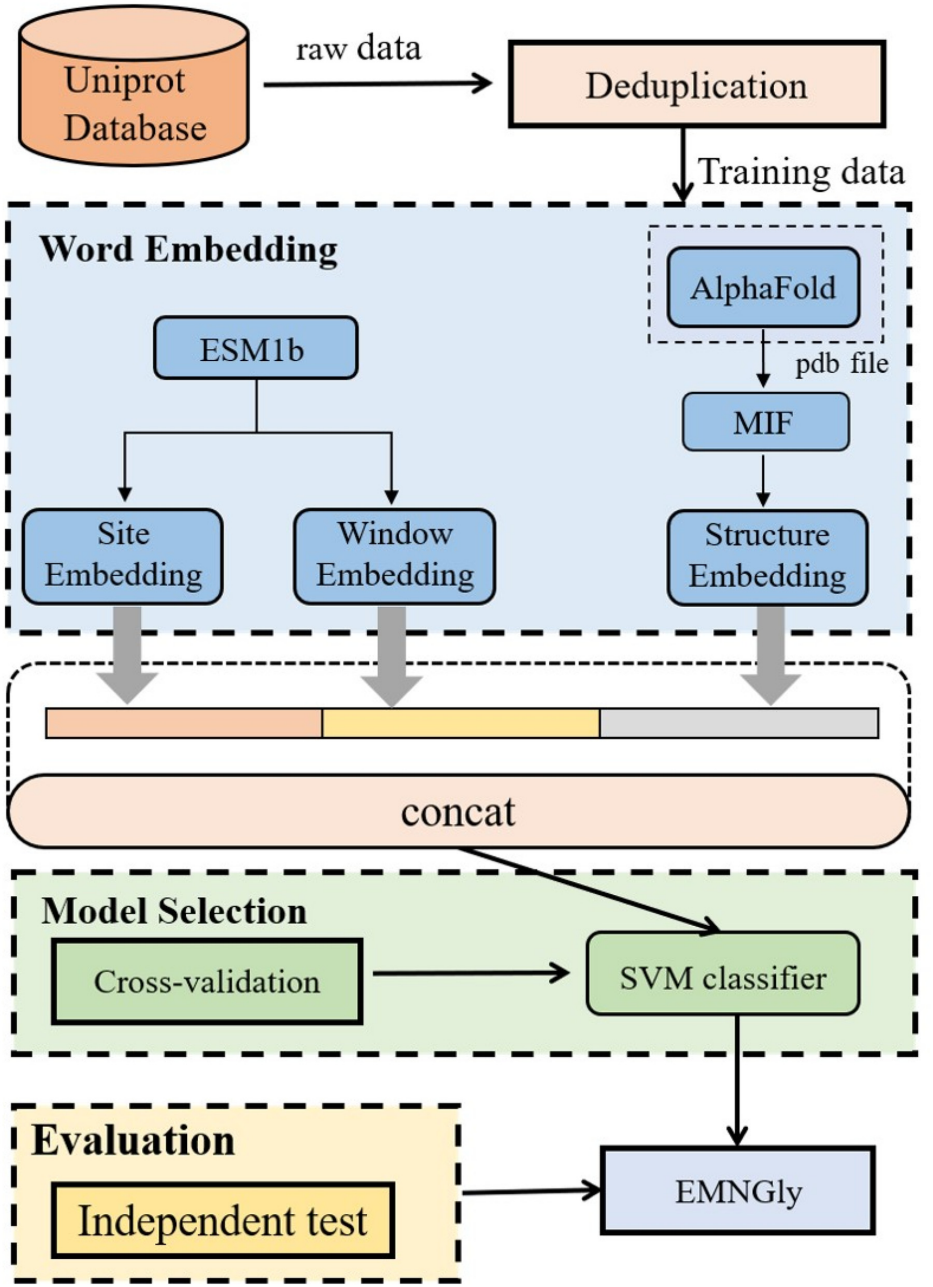
The general framework of EMNGly

The overall framework of our model, EMNGly, depicted in Fig. 1, comprises four stages: data collection and preprocessing, feature embedding, model training, and evaluation.

### 2.1 Datasets

#### 2.1.1 N-GlyDE Dataset

N-GlyDE Dataset, derived from N-GlyDE (Alkuhlani *et al.* 2022), is a glycosylation site annotation dataset that contains information about N-linked glycosylation sites in proteins. It includes experimentally verified glycosylation sites from a variety of species, including human, mouse, rat, and yeast. The dataset provides information on the protein name, UniProt ID, position of the glycosylation site, and the type of glycan attached to the site. It is a valuable resource for researchers studying glycosylation and its role in protein function.

This dataset (Table 1) comprises 2050 confirmed N-glycosylation sites (N-X-[S/T] sequons) from 832 glycoproteins extracted from human Proteins in UniProt (version 201608)  (UniProt Consortium 2015). These sites are considered as positive glycosylation sites since they have been experimentally verified. Furthermore, the dataset includes 1030 sites from the same protein sequences that adhere to the N-X-[S/T] patterns but have not been experimentally verified as N-glycosylation sites. These unverified sites are treated as negative sites. Together, these instances form the training dataset.

**Table 1.** Positive and negative sites for training and testing.

Name of dataset	Positive set		Negative set	
	Training	Independent	Training	Independent
N-GlyDE Dataset	1030	167	2050	280
N-GlycositeAtlas	10 478	1220	10 391	1253

#### 2.1.2 N-GlycositeAtlas

To maximize the amount of data available for training our model, we have integrated multiple additional datasets with DeepNGlyPred, which is currently the largest dataset. The DeepNGlyPred dataset, as described in (Pakhrin *et al.* 2021), contains 12 534 positively annotated sites sourced from N-GlycositeAtlas (Sun *et al.* 2019), paired with 12 534 non-glycosites from the DeepLoc-1.0 dataset. In addition, we have also incorporated benchmark datasets obtained from SPRINT-gly and PUstackgly (Alkuhlani *et al.* 2022).

The combined dataset includes 11 083 glycoproteins, and by removing redundancies and limiting to N-X-[S/T] sequons, it contains 14 869 annotated positive sites and 12 533 non-glycosites.

To prevent overfitting, we took additional measures to eliminate highly similar sequences from the dataset. We removed peptides that shared more than 30% sequence identity using the CD-HIT program (Li and Godzik 2006). As a result, we obtained 11 698 positive and 11 644 negative sites for training and testing our model as shown in Table 1. By using this larger dataset, we ensured that our model was not exclusively trained on closely related peptides, which improves its ability to generalize and perform well on new, previously unseen data.

During the engineering process, we partitioned the dataset by first separating the test set sequences used in LMNglyPred as an independent test set. This set includes 923 protein sequences, consisting of 1220 positive N-linked glycosylation sites and 1253 nonpositive N-linked glycosylation sites. It is exclusively reserved for the final performance comparison with other models. The remaining data is utilized for training, cross-validation, parameter tuning, and model testing.

### 2.2 Glycosylation sites feature representation

To overcome the limitations of manual feature extraction, we have opted to employ deep learning technology for automated feature extraction. Specifically, we utilize the evolutionary scale modeling (ESM) (Rives *et al.* 2021)  pre-trained model for protein sequence feature extraction and the Masked Inverse Folding Model for glycosylation site structure feature extraction. This approach allows us to effectively analyze vast datasets and identify patterns that may be overlooked by manual techniques. By integrating these two methodologies, we can attain a deeper comprehension of the intricate interrelationship between protein sequence and structure and their association with glycosylation.

#### 2.2.1 Evolutionary scale modeling

The first type of feature we employed in this study aims to capture the local and global sequence information present in the amino acid sequences using the ESM model. The ESM model is trained on a large dataset of protein sequences and their evolutionary information, which allows it to capture important features. When used for feature extraction, the model takes a protein sequence as input and produces a vector of numerical features that represent different aspects of the protein’s structure and function.

We utilize the ESM-1b model, consisting of 33 layers and 650M parameters, to extract sequence features and embeddings. This approach allows for a more comprehensive and thorough extraction of protein sequence features, without manual intervention. The model operates by taking in the complete protein sequence as input and creating a 1024-dimensional embedding vector for each amino acid. By using the embedding of the prediction site as a feature representation, we are able to capture sequence information related to all peptides in the sequence, as well as interactions with other amino acids.

To enhance the accuracy of predicting glycosylation, we incorporate a window embedding to supplement the predicted location’s embedding. This is because the amino acids surrounding sequons have a significant impact on glycosylation occurrence at a specific site. We select 20 amino acids before and after the predicted site to create a window with a total length of 41, which enables us to extract local sequence information. For the training dataset, the window size of 41 resulted in the best performance. The feature representation for the entire window is derived from the [CLS] token of the window. In ESM, this token serves as the beginning token in the input sequence, and its fixed-size vector representation is used to represent the entire window sequence.

The PyTorch library and a single A100 GPU are utilized for both training and testing. The processing time for each sequence is around 2 s on an NVIDIA A100 Tensor Core GPU.

#### 2.2.2 Masked inverse folding model

We utilized the MIF (Masked Inverse Folding) (Yang *et al.* 2022) model as the second type of feature to capture structural information present in the protein 3D structure.

Glycosylation sites are typically located on the protein surface or in close proximity to it. Thus, examining the structural profile surrounding an unconfirmed glycosylation site can provide valuable information about its likelihood of being glycosylated. Important factors considered during analysis include nearby amino acids possessing hydroxyl or amide groups that could serve as attachment points for sugar molecules and the degree of solvent accessibility, which impacts enzyme access and glycosylation potential.

Furthermore, structural data can illuminate the chemical and physical characteristics of neighboring residues, including their charge, hydrophobicity, and spatial constraints. This knowledge facilitates precise prediction of glycosylation sites and the identification of the specific sugar moiety involved in binding.

The MIF model serves as a condensed representation of the 3D protein structure at a local level. To achieve this, a masked inverse folding protein language model is used, which is parameterized as a structured graph neural network. For downstream tasks, the input structures are typically required in PDB file format. To obtain the necessary inputs for the MIF model, we retrieved protein PDB files from UniProt. In instances where such files were not available, we utilized AlphaFold2 to acquire them.

The approach employed by the model involves utilizing a structured graph neural network as an encoder to convert the 3D structure of the protein into a compact, low-dimensional representation. This generates a 256-dimensional embedding vector for every site. The embedding vector (256 in length) associated with the site of interest is then used as the structural feature representation for that site. This captures essential information about the local environment, such as the orientation of neighboring residues, hydrogen bonding patterns, and local electrostatic environment. The entire process takes roughly 3 min per protein on an NVIDIA A100 Tensor Core GPU.

### 2.3 Machine learning approaches

This is a classification problem. There are several classification algorithms that can be employed to effectively tackle this issue. Some of the widely utilized classification algorithms include:

1. SVM is a supervised learning algorithm used for classification and regression analysis (Cortes and Vapnik 1995). It works by finding the optimal hyperplane that separates the different classes in a high-dimensional feature space. In cases where the classes are not linearly separable, SVM uses a kernel function to transform the input data into a higher dimensional feature space where the classes may be separable. Some common kernel functions used in SVM are linear, polynomial, radial basis function, and sigmoid.
2. Logistic Regression (Tolles and Meurer 2016) is a statistical learning algorithm used for binary classification problems, where the output variable can take one of two values. It is a type of supervised learning algorithm that models the probability of the outcome based on one or more predictor variables.
3. RF is an ensemble learning method that works by constructing a multitude of decision trees at training time and outputting the class that is the mode of the classes (classification) or mean prediction (regression) of the individual trees.
4. Extreme Gradient Boosting (XGBoost) (Tianqi and Carlos 2016) is a popular and powerful supervised learning algorithm used for both classification and regression tasks. It is an ensemble learning method that combines the predictions of several individual decision trees to produce a final prediction.
5. Gradient Boosting Decision Tree is a machine learning algorithm that belongs to the ensemble learning family. It works by iteratively adding decision trees to a model and improving the model’s prediction by minimizing the loss function through gradient descent optimization.
6. Multi-Layer Perceptron is a type of feedforward artificial neural network that is widely used for both classification and regression tasks. It is composed of multiple layers of interconnected nodes, each of which performs a weighted sum of the inputs and applies a non-linear activation function to produce an output.

All of these models were trained and tested on the dataset using cross-validation. The performance metrics for each model can be found in Supplementary Tables S1 and S3.

### 2.4 Evaluation metrics

To evaluate the performance of our models, we adopted the following four measures: SP, SN, Accuracy, and MCC, which are defined as follows:


(1) $$\text{Specificity}=\frac{\text{TN}}{\text{TP}+\text{FN}}$$

(2) $$\text{Sentivity}=\frac{\text{TP}}{\text{TP}+\text{FN}}$$

(3) $$\text{Accuracy}=\frac{\text{TP}+\text{TN}}{\text{TP}+\text{FP}+\text{TN}+\text{FN}}$$

(4) $$\text{MCC}=\frac{\text{TP}\times \text{TN-FP}\times \text{FN}}{\sqrt{\left(\text{TP}+\text{FP})\times (\text{TP}\times \text{FN})\times (\text{TN}+\text{FP})\times (\text{TN}\times \text{FN}\right)}}$$

where TP represents true positives, TN represents true negatives, FP represents false positives, and FN represents false negatives.

Specificity refers to the ability of a test or measurement to accurately identify the negative samples, as shown in Equation (1). And specificity is often used in combination with sensitivity, which measures the ability of a test to correctly distinguish positive samples, as shown in Equation (2). Accuracy is a measure of how close a measured value is to the actual or true value, as shown in Equation (3). Specificity, specificity, and accuracy are typically expressed as a percentage or a decimal value between 0 and 1. Additionally, MCC is commonly used to evaluate the performance of classification models, particularly in cases where the classes are imbalanced or the dataset is biased, as shown in Equation (4). It takes into account the true positives, true negatives, false positives, and false negatives of a confusion matrix to provide a value between −1 and 1. A score of 1 indicates perfect prediction, 0 indicates random prediction, and −1 indicates total disagreement between prediction and true labels.

The models were measured using 10-fold cross-validation on the training dataset and specifying an independent test set from the beginning before features representation.

## 3 Results

### 3.1 Model performance

We select SVM as the classifier in our final model (the classifier selection process is descripted in Section 3.2).

#### 3.1.1 Performance on the N-GlyDE dataset

Despite its small size, N-GlyDE is a highly popular dataset for benchmarking traditional machine learning methods and has been a primary dataset in protein glycosylation prediction before the rise of deep learning techniques. Thanks to its unique features and manageable size, N-GlyDE is frequently utilized to assess the effectiveness of novel machine learning algorithms and compare their performance against both traditional and deep learning approaches. Furthermore, N-GlyDE has proven to be a valuable tool for investigating the influence of various feature selection methodologies and classifiers, establishing itself as a standard benchmark dataset within the field of protein glycosylation prediction.

To assess the performance of EMNGly against other models, we trained our model on the N-GlyDE training set and applied it to predict N-linked glycosylation sites on the N-GlyDE independent test set and obtained the performance results in terms of measures such as MCC, specificity, sensitivity, and accuracy.

To provide a comprehensive evaluation, we also assessed the performance of established N-linked glycosylation site predictors—LMNglyPred, N-GlyDE, GlycoMine, NetNGlyc, and GlycoEP_Std_PPP—in Table 2. It is worth noting that we employed the same dataset for training EMNGly as the other methods, utilizing site embedding and window embedding generated by ESM-1b of the glycosylated or non-glycosylated token “N” and its neighboring amino acids. The comparison results between our model and the six other tools are presented in Table 2.

**Table 2.** Prediction performance of EMNGly compared to other available N-linked glycosylation site predictors on the N-GlyDE dataset.

Model	MCC	Specificity	Sensitivity	Accuracy	AUC
EMNGly^a^	**0.736**	0.70	**0.975**	**0.884**	**0.946**
LMNglyPred^a^	0.717	**0.747**	0.942	0.869	0.927
DeepNGlyPred^a^	0.605	0.739	0.886	0.794	0.907
N-GlyDE	0.499	0.689	0.826	0.74	0.822
GlycoMine	0.430	0.739	0.700	0.725	0.741
NetNGlyc	0.265	0.411	0.844	0.572	0.668
GlycoEP_Std_PPP	0.119	0.610	0.512	0.574	0.577

a The models are trained on N-GlyDE training dataset.

Note: These approaches are evaluated on each N restricted to N-X-[S/T] sequons in a sequence.

EMNGly achieved an MCC of 0.736, compared to an MCC of 0.717 for LMNglyPred and 0.499 for N-GlyDE. The results in Table 2 indicate that the performance of the EMNGly method is significantly better than all other methods, except for slightly lower specificity compared to LMNglyPred.

The EMNGly, LMNglyPred, and DeepNGlyPred algorithms are categorized as deep learning algorithms, while the remaining four belong to traditional machine learning. The results clearly indicate that deep learning algorithms generally outperform their traditional counterparts. This can be attributed to the superior learning capabilities of deep learning models and the utilization of pre-trained protein models for feature extraction, which provides a significant advantage over other techniques. Despite the vast range of proteins, the available dataset for glycosylation site is still relatively limited in scope. Nevertheless, pre-trained protein models have acquired the ability to encode distributional patterns of amino acids in protein sequences using a large amount of data, which can then be transferred to new tasks using only a minimal amount of annotated data. This facilitates the acquisition of valuable and generalizable features for specific tasks like predicting glycosylation sites, without requiring as much task-specific training data as traditional machine learning methods. Additionally, pre-trained models are capable of capturing complex relationships and patterns within protein sequences that may be challenging to specify manually, thereby potentially enhancing prediction accuracy. Regarding EMNGly, it achieved better results compared to LMNglyPred and DeepNGlyPred by fully and rationally utilizing the embedded information of protein language models.

It should be noted that most of these approaches are evaluated on each asparagine in a protein without being restricted to the N-X-[S/T] sequon, although it has been known for some time that the presence of this consensus sequon does not always result in glycosylation. This could be why the predictive performances of various approaches for predicting N-linked glycosylation sites tend to be high, as the task is simply to predict each in the N-X-[S/T] sequon as a glycosylation site (Pakhrin *et al.* 2021).

#### 3.1.2 Performance on the N-GlycositeAtlas Dataset

Upon analyzing the N-GlyDE Dataset, we discovered that deep learning-based models outperformed other models by a considerable margin. Moving forward, we proceeded to assess the performance of EMNGly, LMNglyPred, and DeepNGlyPred on a larger dataset, specifically the N-GlycositeAtlas dataset. Due to their unsuitability for large-scale datasets, we excluded other models from this analysis.

All three models underwent training using the N-GlycositeAtlas training set and were evaluated on the independent test set of N-GlycositeAtlas. Four indicators, namely MCC, specificity, sensitivity, and accuracy, were measured to determine their performance. Table 2 presents the performance metrics obtained by all models.

EMNGly achieved an MCC of 0.804, compared to an MCC of 0.773 for LMNglyPred and 0.520 for N-GlyDE. The results in Table 3 indicate that the performance of the EMNGly method is significantly better than other two methods, except for slightly lower sensitivity compared to LMNglyPred.

**Table 3.** Prediction performance of EMNGly compared to other available N-linked glycosylation site predictors on the N-GlycositeAtlas set.

Model	MCC	Specificity	Sensitivity	Accuracy	AUC
EMNGly^a^	**0.804**	**0.928**	0.875	**0.902**	**0.976**
LMNglyPred^a^	0.773	0.896	**0.877**	0.887	0.965
DeepNGlyPred	0.520	0.823	0.682	0.753	–

a The models are trained on N-GlycositeAtlas training dataset.

Note: These approaches are evaluated on each N restricted to N-X-[S/T] sequons in a sequence.

This experiment result further confirms that our network architecture is superior to DeepNGlyPred and the current SOTA method LMNglyPred.

### 3.2 Model selection

#### 3.2.1 10-fold cross-validation on the training set

To optimize the hyperparameters responsible for controlling the learning process and evaluate the effectiveness of various deep learning and machine learning models on the training dataset, we conducted a 10-fold cross-validation analysis on the N-GlycositeAtlas dataset. The SVM model yielded the following metrics during the stratified 10-fold cross-validation: MCC (0.804), specificity (0.928), sensitivity (0.875), and accuracy (0.902). Given its favorable performance during cross-validation, we designated this architecture as our final model (see Supplementary Tables S1 and S2 for the complete model selection process).

#### 3.2.2 Testing on the 10% independent test set separated from the N-GlycositeAtlas training set

To evaluate the effectiveness of our approach on an unrelated test set, we trained the model using the complete N-GlycositeAtlas training set and utilized it to predict N-linked glycosylation sites in proteins within an independent test set that was separated from the main N-GlycositeAtlas dataset. The number of samples in each set is presented in Supplementary Table S3. Our results yielded MCC, specificity, sensitivity, accuracy of 0.828, 0.934, 0.893, and 0.914 respectively.

### 3.3 Ablation study

We know that site embedding is the most important feature used in modeling glycosylation sites prediction, and it is also the main feature used by LMNglyPred. In our model, we added two important features: window embedding and local structure embedding, which are used to further characterize the sequence information around glycosylation sites and the spatial information of glycosylation sites in 3D structures. To determine whether these two features are really meaningful, we conducted ablation experiments to test their contributions.

**Figure 2.**
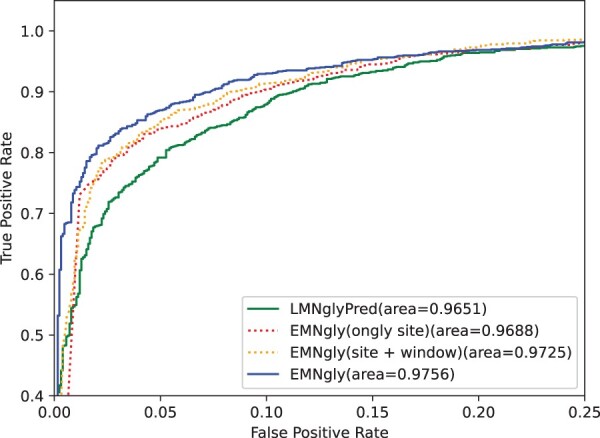
ROC curves of LMNglyPred, EMNgly(site embedding, site+window embedding), EMNgly on the independent dataset. For each model, the area under the ROC curve is reported

The experimental results shown in Table 4 and Fig. 2 indicate that when only using site embedding feature, the model has already achieved good performance, with an MCC value of 0.788. With the addition of window embedding and local structure embedding, the model performance gradually improves, with MCC values reaching 0.794 and 0.804, respectively. The experimental results indicate that site embedding feature is indeed one of the most important features in glycosylation site prediction, but the addition of window embedding and local structure embedding features can further improve the model’s performance. This suggests that in glycosylation site prediction, not only the sequence information around the site needs to be considered but also the 3D structural environment where the site is located, and these information are of great significance for improving the model’s performance. Therefore, considering multiple features is very important in glycosylation site prediction.

**Table 4.** Ablation study of our approach on the training dataset.

Features	MCC	Specificity	Sensitivity	Accuracy
site	0.788	0.903	0.885	0.894
site+window	0.794	0.894	0.903	0.898
site+window+structure	0.804	0.928	0.875	0.902

site: site embedding; window: window embedding; structure: local structure embedding.

## 4 Discussion

We have created a state-of-the-art glycosylation site predictor, EMNGly, that utilizes protein language models and advanced deep learning techniques. Our innovative approach has outperformed existing tools in accurately predicting glycosylation sites, showcasing exceptional performance.

To minimize overfitting and enhance the precision of our models, we have compiled a dataset for glycosylation sites by merging information from three distinct data sources. Nevertheless, there exists a scarcity of databases relevant to glycosylation.

And it remains a challenge to accurately identify non-glycosylation or negative sites. Based on the reviewed studies, it appears that non-glycosylation (negative) sites are largely unlabeled sites. The acquisition of definitive non-glycosylated site data is very difficult, mainly due to the lack of experiments confirming non-glycosylated sites, which can result in a biased prediction model.

Recently, several protein language models (PLMs) have been proposed to obtain representations from large collections of protein sequences. The development of these PLMs has opened up unprecedented opportunities for protein research to predict the structure, function, and interactions of proteins (Pokharel *et al.* 2022, Thumuluri *et al.* 2022). In particular, the application of PLMs has also attracted widespread attention for the study of protein glycosylation sites. In the future, we can expect that PLMs will play an increasingly important role in the field of glycoproteomics, where the identification and characterization of protein glycosylation sites are crucial for understanding the function of many proteins.

**Figure 3.**
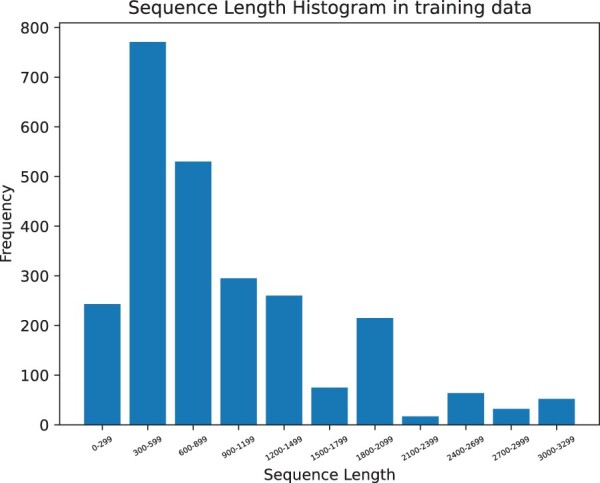
Length distribution of Independent dataset regenerated

**Figure 4.**
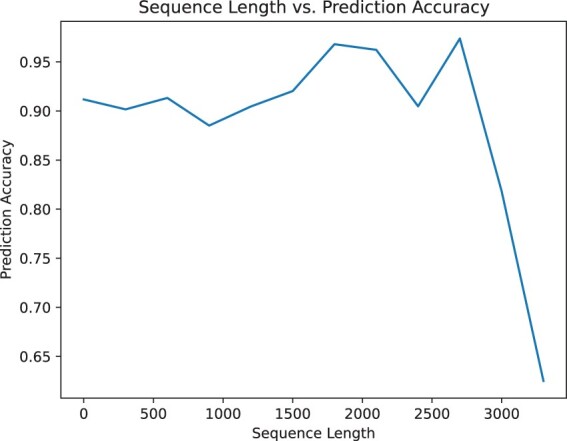
Change in accuracy based on the length of the protein sequences computed on the independent dataset

Additionally, predicting glycosylation sites is related to the length of the protein sequence. Figs 3 and 4 illustrates that our model can still achieve good prediction accuracy on long protein sequences.

Key points

- We have created a state-of-the-art glycosylation site predictor, EMNGly.
- We utilizes protein language models and advanced deep learning techniques for feature extraction.
- The model integrates structural information, including the surrounding environment of the glycosylation site and the physical and chemical properties of residues.
- we have compiled a dataset for glycosylation sites by merging information from three distinct data sources.

The test code and data of EMNgly are available online at https://github.com/StellaHxy/EMNgly.

## Supplementary Material

btad650_Supplementary_Data
