# Supplementary material for: EMNGly: predicting N-linked glycosylation sites using the language models for feature extraction
Source: Bioinformatics. 2023 Nov 1;39(11):btad650. doi: 10.1093/bioinformatics/btad650 (PMC10627407; doi:10.1093/bioinformatics/btad650)
Supplement: btad650_Supplementary_Data [file btad650_supplementary_data.docx]

**EMNGly: predicting N-linked glycosylation sites using the language models for feature extraction**

**Supplementary Material**

**SUPPLEMENTAL RESULTS**

**3.2.1**

To optimize the hyperparameters, which are responsible for controlling the learning process, and evaluate the effectiveness of different DL/ML models on the training dataset, we conducted a 10-fold cross-validation analysis on the N-GlycositeAtlas dataset.

We not only conducted tests on commonly used single classifiers, but also on various ensemble classifiers. Additionally, to prevent data leakage and obtain accurate predictive performance from our models, we ensured that different sites within the same protein sequence were not present in both the training and validation sets during cross-validation.

Table S1 displays the predictive performance of various DL and ML models.

The pre-trained ESM-1b model generates contextualized embedding for the token "N" in each sequon and window embedding for the sequon along with its neighboring amino acids, which, upon being fed to SVM, yields the most superior performance.

The SVM model yielded the following metrics for the stratified 10-fold cross-validation: MCC (0.804), Specificity (0.928), Sensitivity (0.875), and Accuracy (0.902). As it produced the most favorable outcome during cross-validation, we designated this architecture as our ultimate model-EMNGly. Notably, the independent test set result corroborated the 10-fold cross-validation findings, Table S1, indicating that EMNGly is a reliable tool for N-linked glycosylation prediction.

Table S2 shows the optimal value for each parameter for the six classifiers.

**Table S1.** Results of the 10-fold cross-validation on the training dataset using different deep and machine learning models.

| **Model** | **MCC** | **Specificity** | **Sensitivity** | **Accuracy** |
| --- | --- | --- | --- | --- |
| **LR** | 0.7929 | 0.9025 | 0.8901 | 0.8964 |
| **RF** | 0.7241 | 0.8765 | 0.8470 | 0.8619 |
| **XGBoost** | 0.7785 | 0.8990 | 0.8792 | 0.8891 |
| **GDBT** | 0.7591 | 0.8988 | 0.8596 | 0.8793 |
| **MLP** | 0.7975 | 0.8915 | 0.9054 | 0.8984 |
| **SVM*** | 0.788 | 0.9033 | 0.8851 | 0.8940 |
| **SVM** | 0.8042 | 0.9279 | 0.8754 | 0.9016 |

* Only site embedding

**Table S2.** The optimal value for each parameter for six classifiers.

| **Classifier** | **Parameter** | **Optimal Value** |
| --- | --- | --- |
| **LR** | Penalty | L2 |
| **RF** | n_estimators | 100 |
| **XGBoost** | n_estimators | 100 |
| **GDBT** | n_estimators | 100 |
| **MLP** | solver | adam |
|  | hidden_layer_sizes | (1280,1280) |
| **SVM** | kernel | rbf |
|  | C | 100 |
|  | Gamma | 1e-4 |

**3.2.2**
**Table S3**. Performance metrices of various machine and deep learning models on the independent test dataset separated from the training dataset.

| **Model** | **MCC** | **Specificity** | **Sensitivity** | **Accuracy** |
| --- | --- | --- | --- | --- |
| **LR** | 0.8202 | 0.9146 | 0.9051 | 0.9101 |
| **RF** | 0.7441 | 0.8937 | 0.8542 | 0.8721 |
| **XGBoost** | 0.8023 | 0.9109 | 0.8924 | 0.9012 |
| **GDBT** | 0.7687 | 0.9026 | 0.8652 | 0.8842 |
| **MLP** | 0.8223 | 0.8953 | 0.8918 | 0.9012 |
| **SVM*** | 0.8070 | 0.9154 | 0.8921 | 0.9032 |
| **SVM(EMNGly)** | 0.8282 | 0.9343 | 0.8934 | 0.9143 |

* Only site embedding
